# Fabrication of Silicon Nanobelts and Nanopillars by Soft Lithography for Hydrophobic and Hydrophilic Photonic Surfaces

**DOI:** 10.3390/nano7050109

**Published:** 2017-05-11

**Authors:** Estela Baquedano, Ramses V. Martinez, José M. Llorens, Pablo A. Postigo

**Affiliations:** 1 Instituto de Microelectrónica de Madrid, CSIC, Tres Cantos, 28760 Madrid, Spain; estela.baquedano@csic.es (E.B.); jose.llorens@imm.cnm.csic.es (J.M.L.); 2 School of Industrial Engineering, Purdue University, 315 N. Grant Street, West Lafayette, IN 47907, USA; rmartinez@purdue.edu; 3 Weldon School of Biomedical Engineering, Purdue University, 206 S. Martin Jischke Drive, West Lafayette, IN 47907, USA

**Keywords:** soft lithography, photonic surface, hydrophobic, hydrophilic, silicon nanobelts

## Abstract

Soft lithography allows for the simple and low-cost fabrication of nanopatterns with different shapes and sizes over large areas. However, the resolution and the aspect ratio of the nanostructures fabricated by soft lithography are limited by the depth and the physical properties of the stamp. In this work, silicon nanobelts and nanostructures were achieved by combining soft nanolithography patterning with optimized reactive ion etching (RIE) in silicon. Using polymethylmethacrylate (PMMA) nanopatterned layers with thicknesses ranging between 14 and 50 nm, we obtained silicon nanobelts in areas of square centimeters with aspect ratios up to ~1.6 and linewidths of 225 nm. The soft lithographic process was assisted by a thin film of SiO*_x_* (less than 15 nm) used as a hard mask and RIE. This simple patterning method was also used to fabricate 2D nanostructures (nanopillars) with aspect ratios of ~2.7 and diameters of ~200 nm. We demonstrate that large areas patterned with silicon nanobelts exhibit a high reflectivity peak in the ultraviolet C (UVC) spectral region (280 nm) where some aminoacids and peptides have a strong absorption. We also demonstrated how to tailor the aspect ratio and the wettability of these photonic surfaces (contact angles ranging from 8.1 to 96.2°) by changing the RIE power applied during the fabrication process.

## 1. Introduction

Vertical nanostructures with aspect ratios >1 are essential in a variety of fields such as photonics and nanophotonics [1,2], microfluidics [3,4], microelectromechanical systems (MEMs) [5] and even in lab-on-a-chip bio-applications such as DNA separation [6]. Soft lithography was introduced as a low-cost alternative to conventional lithography [7] and has been shown to be a powerful method of generating reproducible nanopatterns and nanostructures with features sizes ranging from 30 to 100 µm [8] using elastomeric stamps made of polydimethylsiloxane (PDMS). Unfortunately, the low Young’s modulus of elastomeric formulations (such as PDMS, h-PDMS, and PTFE) limits the aspect ratio of the nanostructures that can be achieved using soft lithography [9]. When the aspect ratio is too high or too low, the nanostructures on the PDMS stamp tend to deform and produce defects in the patterned areas due to the pressure exerted on the stamp during the patterning process [8,9,10]. Nanoimprint lithography (NIL) is also affected by this limitation, requiring complex post-processing steps to achieve deep nanostructures [11]. 

Several top-down methods have been developed to enable the fabrication of high-aspect-ratio nanopatterns. Some methods use a double layer of resist and a thin film like hard mask among the layers to increase the high aspect ratio in the bottom resist [10,12]. However, for transferring the pattern into Si, it is always necessary to continue the process with a subsequent etching [7]. In this work, a process without a second layer resist is developed through the optimization of the reactive ion etching (RIE) process to transfer the pattern into Si. The RIE process uses only SF_6_, CHF_3_, and a SiO*_x_* mask in a standard RIE chamber, avoiding any cryogenic or other deep-RIE procedures. Using this patterning-etching procedure, we obtained gratings made out of nanobelts with aspect ratios >1 using conventional DVD and Blu-ray discs as soft lithographic masters in a simple and low-cost approach [13,14,15].

RIE is a reliable method to achieve high-aspect-ratio silicon nanostructures [16] that can use a mixture of diverse gases such as O_2_:Ar or Cl_2_ [10,17]. SF_6_ plasma is commonly used as the main reactive agent to etch silicon due to its capacity to generate atomic fluorine (F), which has a high affinity for Si atoms to form SiF*_x_* (*x* = ¼, 2, 4) volatile species [18,19]. The addition of small concentrations of O_2_ in the SF_6_ plasma leads to the generation of F due to the following oxidation reaction: O + SF*_x_* → SOF*_x_*_−1_ + F (*x* ≤ 5) [18]. To our knowledge, the best aspect ratios (as high as 107) have been achieved using deep RIE (DRIE) [20], and values of 50 have been reported using Inductively Coupled Plasma (ICP-RIE) [21]. Unfortunately, the DRIE and ICP-RIE systems are not as extended as conventional RIE systems due to their higher price. In general, using conventional RIE systems, it is difficult to achieve aspect ratios larger than 10 and lateral dimensions smaller than 200 nm when using Ni-masks and electron-beam lithography on thicker PMMA layers (around 300 nm) [22]. This manuscript describes the cost-effective fabrication of nanostructures 535-nm-tall and 197-nm-diameter (aspect ratio 2.71) over large areas using a conventional RIE system and starting from PMMA layers as thin as 14 nm. This simple fabrication process has two steps: (i) soft lithography is used to pattern large silicon surfaces with PMMA nanostructures; (ii) the PMMA nanostructures are used as a mask during the RIE etching of a sacrificial SiO*_x_* layer that is 14 nm thick. We varied the RIE power applied to SF_6_/O_2_ plasma to find the etching conditions that optimized the aspect ratio of the resulting nanostructures and to control the wetting properties of the final photonic surfaces. 

## 2. Experiment

**Figure 1:**
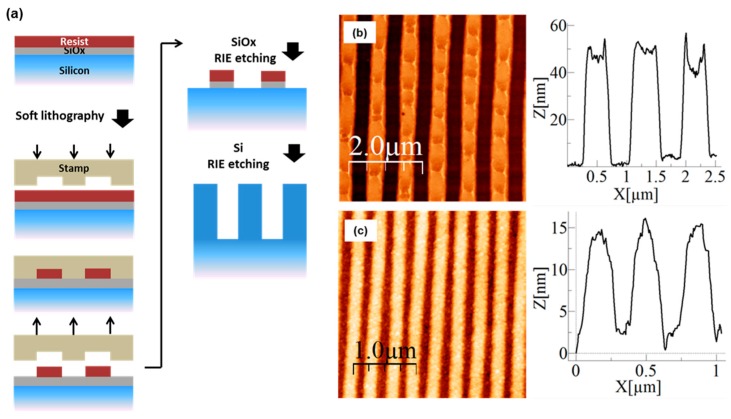
(**a**) Schematic illustration of the fabrication process; (**b**) AFM image of the nanopattern obtained by soft lithography using DVD stamp; (**c**) a nanopattern obtained by soft lithography using a Blu-ray (BR) stamp.

Figure 1a shows the fabrication process sequence. First, a thin layer of SiO*_x_* ~14 nm thick was deposited on a Si wafer to serve as a hard mask by a plasma-enhanced chemical vapor deposition (PECVD) system (Surface Technology Systems 310PC-DF) at 300 °C. We fabricated elastomeric stamps using the following masters: (i) digital versatile discs (DVDs) with a lineal grating of 775 nm period, a 400 nm linewidth, and a 150 nm depth. DVDs were replicated by casting a 5 mm layer of Sylgard 184 PDMS (Dow Corning Corporation, Seneffe, Belgium); (ii) Blu-ray (BR) discs with a lineal grating of 325 nm, a 200 nm linewidth, and a 25 nm depth. We replicated BR discs using a composite formed by a stiff layer of h-PDMS (30–40 µm thick) supported by a flexible layer (5 mm thick) of Sylgard 184 PDMS [23]. The patterning process over the Si/SiO*_x_* substrate required 5 µL of resist (5% PMMA 996k in gamma-butyrolactone (GBL)) added with a micropipette. The resist solution was covered with the PDMS stamp and pressed between two glass slides. The resist was cured under vacuum for 3 h. Figure 1b,c shows the atomic force microscope (AFM) images of the nanopatterns obtained in the PMMA. The depth of the nanopattern was 50 nm using the DVD stamp (DVD-PDMS sample). For the sample fabricated with a BR stamp (BR-hPDMS sample), the depth was 15 nm. The limiting factor for a higher aspect ratio is the thickness of SiO*_x_*, which can be etched using the PMMA. Using our etching recipe, this thickness is between 14 and 20 nm. After that, two RIE etchings were carried out (Oxford Plasmalab 80). A cleaning procedure of the RIE chamber with Ar and O_2_ was run before each etching to ensure reproducibility. The first etching (CHF_3_ 25.0 sccm, chamber pressure 20.0 mTorr, power 50 W, wafer temperature 30 °C) was used to transfer the nanopattern to the SiO*_x_* hard mask. The second etching (SF_6_/O_2_ 12.0/3.0 sccm, chamber pressure 90.0 mTorr, wafer temperature 30 °C, 1 min) was used to transfer the nanopattern to the Si [24]. We varied the RIE power between 10 and 100 W to explore its effect in the aspect ratio, while keeping the rest of the parameters fixed. Each experiment was carried out three times in order to verify its reproducibility.

## 3. Results and Discussion

### 3.1. Optimization of the RIE Process

**Figure 2:**
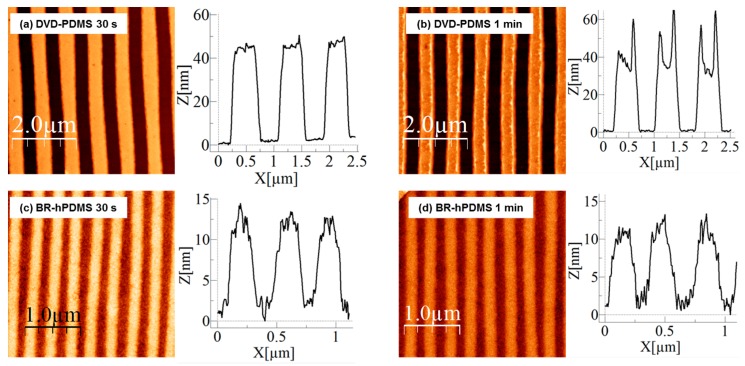
AFM images after reactive ion etching (RIE) etching of a 14-nm-thick SiO*_x_* layer with CHF_3_. (**a**) AFM image of DVD-PDMS sample after 30 s of etching with CHF_3_ plasma; (**b**) AFM image of DVD-PDMS sample after 1 min of RIE etching with CHF_3_ plasma; (**c**) AFM image of BR-hPDMS sample after 30 s of RIE etching with CHF_3_ plasma; (**d**) AFM image of BR-hPDMS sample after 1 min of RIE etching with CHF_3_ plasma.

We determined first the etching time necessary to transfer the PMMA nanopattern to the 14-nm-thick SiO*_x_* by AFM inspection. During the RIE process, SiO*_x_* and PMMA were etched at the same rate. The selectivity between PMMA and SiO*_x_* is 1:1. Figure 2 shows that 30 s were not enough time to completely transfer the nanopatterns onto the SiO*_x_* surface (DVD-PDMS samples). After a 1 min RIE process, the nanopatterns were transferred over large areas of the SiO*_x_* surface. BR-hPDMS samples transferred the nanopatterns after 30 s; however, since 1 min of etching did not change the nanopattern significantly, we decided to use 1 min for both samples. 

**Figure 3:**
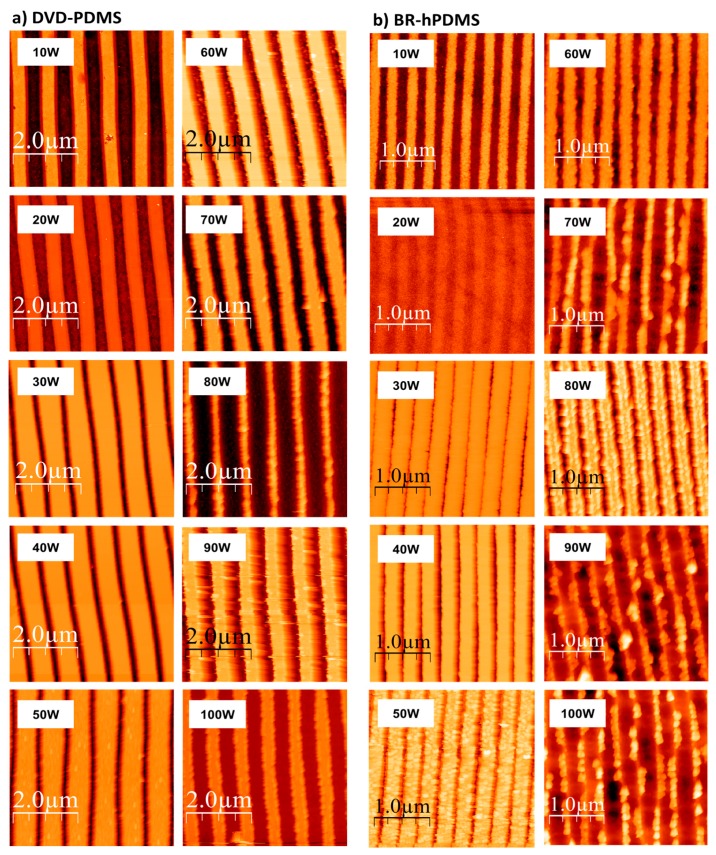
AFM images after RIE with SF_6_/O_2_ mixture plasma for different RIE powers. The images on the left correspond to DVD-PDMS samples, while those on the right were obtained using BR-hPDMS stamps.

We also studied the influence of the RIE power during the etching of the Si wafer. Increasing the RIE power produces more reactive species and improves the ionic bombardment. This affects the etching rate and both the width and roughness of the patterned nanostructures. SEM pictures show that no artifacts like “grass” or any other high-level roughness is present (Figure S1). Ten different RIE powers were used starting from 10 to as much as 100 W. The etching time was fixed at 1 min. Figure 3 shows the AFM images of the nanopatterns obtained after the etching (Figure S2).

Table 1 and Table 2 show the results for DVD-PDMS and BR-hPDMS samples, respectively. The results were the average of the three experiments for each RIE power. 

**Figure 4:**
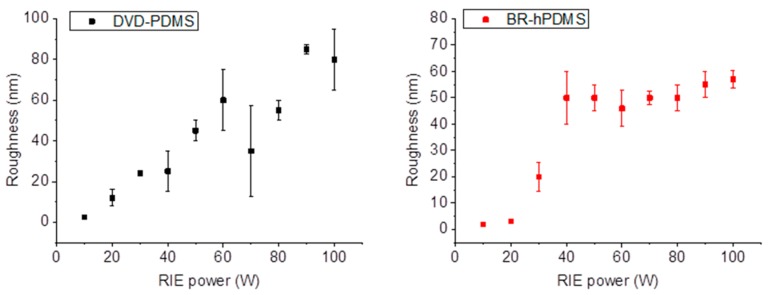
Dependence of the roughness of the nanostructures fabricated on the RIE power used for DVD-PDMS and BR-hPDMS samples.

When the RIE power was increased, the surface roughness also increased (Figure 4) for both kinds of samples with a maximum value of 87.5 nm at 90 W in the case of DVD-PDMS and 60 nm at 80 W in BR-hPMDS. 

**Figure 5:**
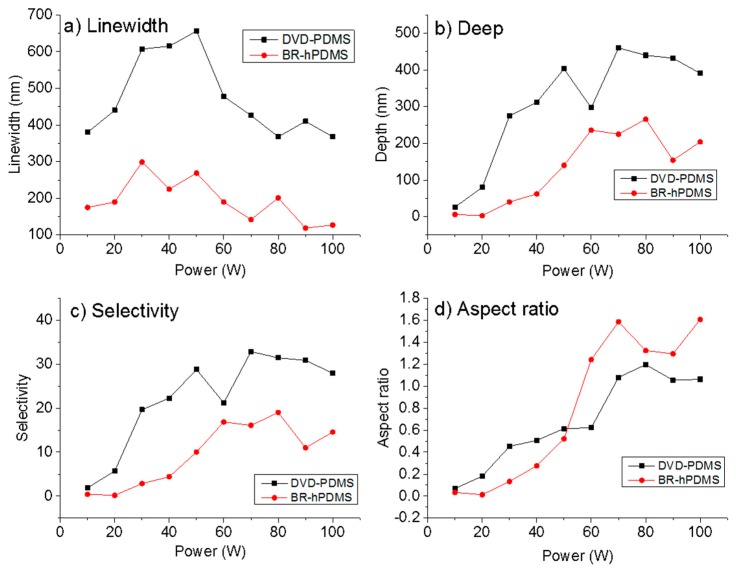
(**a**) Variation of linewidth versus the RIE power in the SF_6_/O_2_ plasma etching. (**b**) Variation of depth with the RIE power in the SF_6_/O_2_ plasma etching. (**c**) Change in the selectivity to SiO*_x_*/Si with the RIE power in the SF_6_/O_2_ plasma etching. (**d**) Aspect ratio versus the RIE power in the SF_6_/O_2_ plasma etching.

Figure 5 shows the change in linewidth, depth, selectivity, and aspect ratio versus the RIE power. Figure 5a shows an increase in the linewidth when the RIE power is lower than 70 W for DVD-PDMS samples. Higher RIE powers maintain the linewidth with respect to the original linewidth (previous to the RIE process). In the case of BR-hPDMS samples, the change in the linewidth is less abrupt. That could be due to the increased difficulty for the reactive species to penetrate between the patterned lines during the etching. Figure 5b shows the variation of the depth with the RIE power for both sets of samples. As the RIE power increases, the depth of the nanostructures is higher for the DVD-PDMS samples. This is not related to the initial thickness of the PMMA resist but rather with the geometry and dimensions of the stamp used. The maximum depth achieved is 460 nm at 70 W. For the BR-hPDMS samples, the maximum depth achieved is 266 nm at 80 W. For higher RIE powers, the SiO*_x_* mask is overetched. This produces a drop in the Si depth that can be etched. Figure 5c shows the selectivity (SiO*_x_*/Si) for the SF_6_/O_2_ etching at different RIE powers. In the DVD-PDMS samples, the highest selectivity of 1:32.8 is achieved at 70 W. For BR-hPDMS samples, a high selectivity of 1:19 is achieved at 80 W. The aspect ratio (depth/linewidth) is shown in Figure 5d. In the DVD-PDMS samples, the highest aspect ratio is 1.19 obtained at 80 W; in BR-hPDMS, 1.58 at 70 W. 

**Figure 6:**
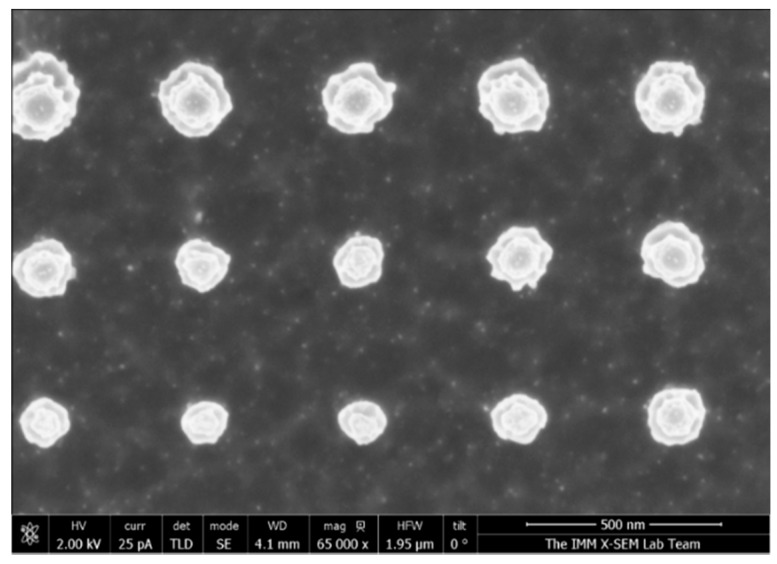
SEM images of 2D nanostructures of silicon fabricated using 70 W of RIE power.

To fabricate 2D arrays of nanostructures (nanopillars), we performed a similar optimization of the etching by varying the RIE power. The rest of the conditions were the same than used for the fabrication of the Si nanobelts. A master with nanoholes of diameter ~150 nm and a period ~400 nm was used to obtain the stamp. Figure 6 shows a SEM image of the obtained nanopattern.

**Figure 7:**
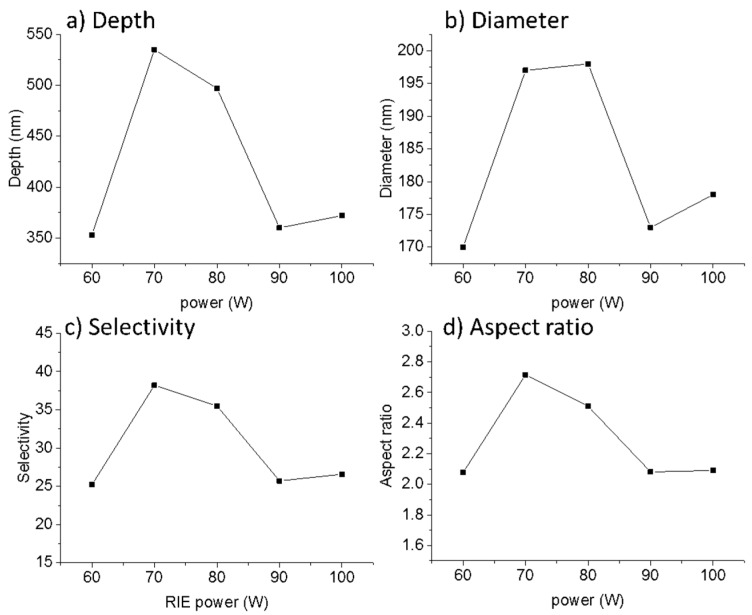
(**a**) Variation of depth versus the RIE power in the SF_6_/O_2_ plasma etching of 2D nanopillars. (**b**) Variation of the diameter versus the RIE power. (**c**) Selectivity (SiO*_x_*/Si) dependence on the RIE power. (**d**) Aspect ratio dependence on the RIE power.

Table 3 shows the dimensions and the selectivity values obtained for 2D nanostructures. Figure 7a shows the relationship between the depth and the RIE power. We obtained a maximum depth of 535 nm at 70 W. Figure 7b shows an increase in the diameter of the nanopillars for RIE powers from 70 to 80 W. We found the highest selectivity of SiO*_x_*/Si to be 1:38.2 at 70 W (see Figure 7c). Figure 7d shows the aspect ratio (depth/diameter) of the nanopillars with a maximum of 2.71 at 70 W of RIE power.

### 3.2. Wettability

**Figure 8:**
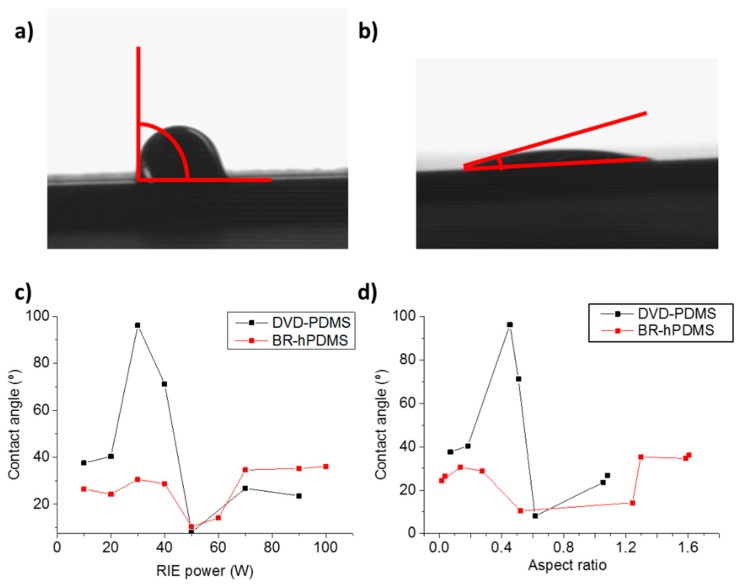
(**a**) Image of the contact angle of a 0.5 µL droplet of water on top of a DVD-PDMS Si nanobelt (etched at 30 W). (**b**) Same for nanobelts etched at 50 W. (**c**) Contact angle versus the RIE power for DVD-PDMS and BR-hPDMS nanobelts. (**d**) Contact angle versus the aspect ratio for DVD-PDMS and BR-hPDMS nanobelts.

Controlling the wetting properties of a variety of surfaces through the manipulation of their physical and/or chemical properties at the nanoscale level has attracted increased attention due to their multiple applications [25]. Hydrophilic surfaces with a water contact angle of almost 0° have been successfully used as a transparent coating with antifogging and self-cleaning properties, while hydrophobic surfaces can avoid contamination, the sticking of snow, and erosion [26]. The modification of the surface morphology and the materials used permits to adjust the wettability from a hydrophilic material to a hydrophobic one [27]. Specific periods and aspect ratios play an important role for obtaining hydrophilic or hydrophobic states [28]. The Wenzel model [29] describes sessile droplets that fully wet the surface texture. On the other hand, the Cassie–Baxter model [30] describes water droplets that reside partially on the solid texture and partially on a raft of air pockets entrapped within the microscopic texture that enable the surface to become hydrophobic. In addition to high aspect ratios (>>1), the feature density, the characteristic geometric length scale, and topography of the surface texture all play pivotal roles in creating hydrophobic surfaces that exhibit robust Cassie–Baxter interfaces and that can resist wetting under dynamic conditions [31,32,33]. Studies have shown that an array of high-aspect-ratio nanostructures with high densities shows hydrophobicity, with strong resistance against transition to the Wenzel state [28,32,34]. We studied the wettability properties of the fabricated Si nanobelt surfaces through the measurement of the contact angle. A 0.5 µL drop of water was placed over the nanostructured surface to measure its contact angle (Figure 8a,b). Figure 8c shows that the highest value for the contact angle for DVD-PDMS samples was 96.2° (etched with 30 W of RIE power) and the lowest value was 8.1° (50 W). For the BR-hPDMS samples, the highest value of the contact angle was 36.1° (100 W) and the lowest value was 10.5° (50 W). We observed that DVD-PDMS samples had a higher hydrophobicity for low RIE powers (<50 W) and BR-hPDMS samples for higher RIE powers (>70 W). However, for both types of samples, a high hydrophilic surface was obtained at 50 W. Figure 8d shows the change in the contact angle with the aspect ratio. In DVD-PDMS, the highest hydrophobicity was achieved at an aspect ratio of 0.45; in BR-hPDMS samples, at 1.6. We found that, for all surfaces, the wetting state corresponds to a Wenzel state and the contact angle never exceeds the critical angle needed for the transition to a Cassey–Baxter condition (Figures S3–S6 and Tables S1–S3).

### 3.3. Optical Measurements

**Figure 9:**
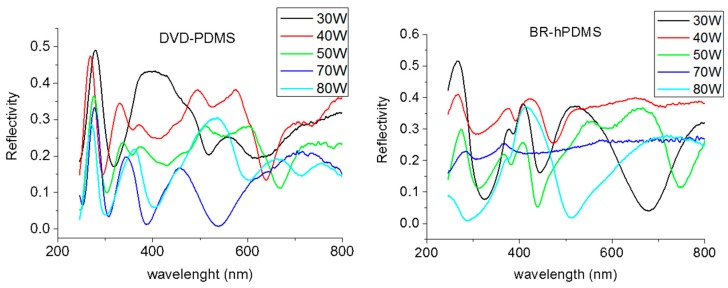
Reflectivity spectrums for the DVD-PDMS and BR-hPDMS samples.

The interaction of materials with optical waves and photons is strongly dependent on the structure of their surface. Nanoscale modifications of the structure of a surface can be used to control the light field distribution and light propagation, allowing for the development of a large range of key components for optical systems [35]. We measured the reflectivity of the silicon nanobelts in the polarizations s and p. Figure 9 shows the reflectivity spectra in the polarization s (polarization p showed lower intensity for both sets of samples) using a UV-visible elipsometer (J.A Woolam M-2000FI, J.A Woolam Co, Lincoln, NE, USA) Measurements were performed at 45° on the DVD-PDMS and BR-hPDMS samples fabricated with different RIE powers. 

For both cases, we obtained high oscillations in the reflectivity with maximum values reaching 50% of reflectivity. The first peak, around 280 nm, is correlated with the high reflectivity peak of silicon, although in our case this peak is much narrower due to the nanostructuration (Figure S7). This is an interesting spectral region in the UVC part of the light spectrum where most proteins show strong optical absorptions [36]. The rest of the peaks were naturally produced by the periodicity of the samples. In the DVD-PDMS samples, as well as in the BR-hPDMS samples, the spectrums showed that the highest reflectivity was achieved when the RIE power was 30 W, and decreased when the RIE power was increased. 

## 4. Conclusions

This research demonstrates a new method for the fabrication of nanogratings and nanopillars onto Si wafers with aspect ratios >1 by combining soft lithography and RIE starting from nanopatterned resist layers as thin as 14 nm. We fabricated 1D and 2D nanostructures using low-cost DVD and BR stamps, over large areas, in a simple, fast, and reproducible way. We demonstrated the control of the aspect ratio of the fabricated silicon nanostructures by varying the RIE power during the RIE process. By optimization of the RIE power, we fabricated nanobelts with aspect ratios up to 1.6 (142 nm of linewidth and 225 nm of depth, using BR-hPDMS stamps) and an aspect ratio of 2.71 for 2D nanopillars (197 nm of diameter and 535 nm of depth). We also demonstrated how the wetting properties of the photonic surfaces that are patterned using this method can be tuned. For the DVD-PDMS samples, the highest angle achieved was 96.2° and the lowest was 8.1°. For BR-hPDMS, the contact angle varied between 36.1 and 10.5°. Finally, the reflectivity was measured with a UV-visible elipsometer, obtaining a narrow and intense peak of intensity in the UVC spectral region (280 nm), where some aminoacids and peptides have a strong optical absorption. 

## Figures and Tables

**Table 1:** Results obtained for the different RIE powers studied in the DVD-PDMS samples.

	RIE Power									
	10 W	20 W	30 W	40 W	50 W	60 W	70 W	80 W	90 W	100 W
**Roughness (nm)**	2.1	8	24.5	35	40	45	57.5	50	87.5	65
**Linewidth (nm)**	380	440	607	615	656	478	426	368	410	368
**Depth (nm)**	26.5	80	275	312	403	297	460	440	432	391
**Selectivity SiO*_x_*/Si**	1.8	5.7	19.6	22.2	28.7	21.2	32.8	31.4	30.85	27.92
**Aspect Ratio**	0.07	0.18	0.45	0.51	0.61	0.62	1.08	1.19	1.05	1.06

**Table 2:** Results obtained for the different RIE powers studied in the BR-hPDMS samples.

	RIE Power									
	10 W	20 W	30 W	40 W	50 W	60 W	70 W	80 W	90 W	100 W
**Roughness (nm)**	2.5	2.5	25.5	60	45	53	47.5	55	50	53.5
**Linewidth (nm)**	175	190	299	225	269	190	142	201	119	127
**Depth (nm)**	5.96	2.52	40	62	140	236	225	266	154	204
**Selectivity SiO*_x_*/Si**	0.42	0.18	2.85	4.42	10	16.85	16.07	19	11	16.57
**Aspect Ratio**	0.03	0.01	0.13	0.27	0.52	1.24	1.58	1.32	1.29	1.61

**Table 3:** Results obtained in 2D nanostructures for different RIE powers.

	RIE Power				
	60 W	70 W	80 W	90 W	100 W
**Diameter (nm)**	170	197	198	173	178
**Depth (nm)**	353	535	497	360	372
**Selectivity SiO*_x_*/Si**	25.2	38.2	35.5	25.7	26.5
**Aspect Ratio**	2.07	2.71	2.51	2.08	2.09

## Supplementary Materials

The following are available online at http://www.mdpi.com/2079-4991/7/5/109/s1.
