# Supplementary material for: Fabrication of Silicon Nanobelts and Nanopillars by Soft Lithography for Hydrophobic and Hydrophilic Photonic Surfaces
Source: Nanomaterials (Basel). 2017 May 11;7(5):109. doi: 10.3390/nano7050109 (PMC5449990; doi:10.3390/nano7050109)
Supplement: Supplementary file 1 [file nanomaterials-07-00109-s001.pdf]

SUPPLEMENTARY INFORMATION

Fabrication of Silicon Nanobelts and Nanopillars by Soft Lithography for Hydrophobic and Hydrophilic Photonic Surfaces

E Baquedano[1], R V Martinez[2, 3], J M Llorens[1], P A Postigo[1]

[1] Instituto de Microelectrónica de Madrid, CSIC, 28760 Tres Cantos, Madrid (Spain)
[2] School of Industrial Engineering, Purdue University, 315 N. Grant Street, West Lafayette, IN 47907 (USA)
[3] Weldon School of Biomedical Engineering, Purdue University, 206 S. Martin Jischke Drive, West Lafayette, IN 47907 (USA)

SEM analysis

SEM images (FEI verios 460) of the nanowire surface for three representative cases. The rest of the samples show a similar appearance under the SEM.

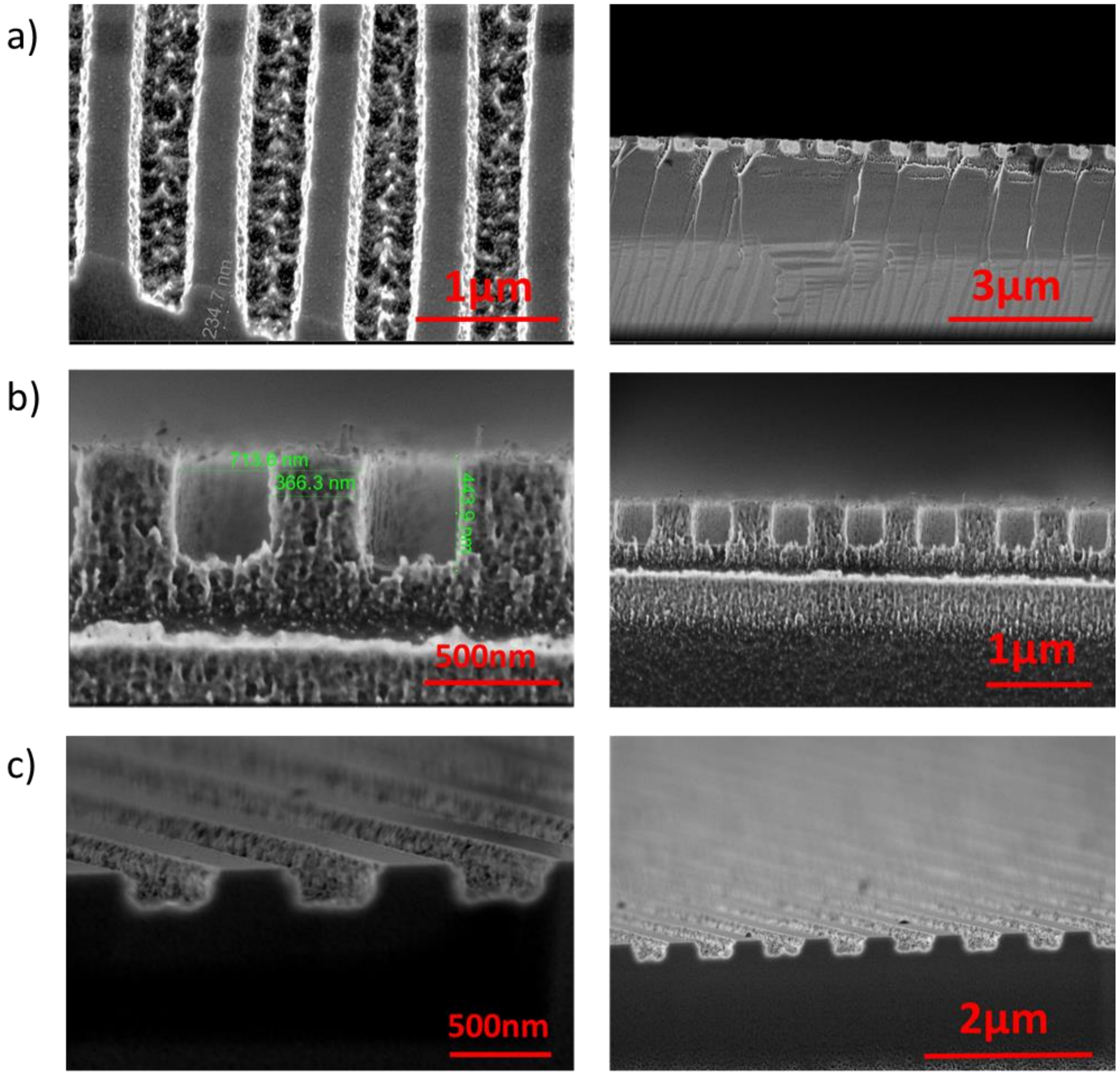

Figure S1. SEM pictures of nanowires. a) DVD-PDMS at 60 W b) DVD-PDMS at 70 W c) DVD-PDMS at 90 W

AFM profiles corresponding to the AFM images Figure 3

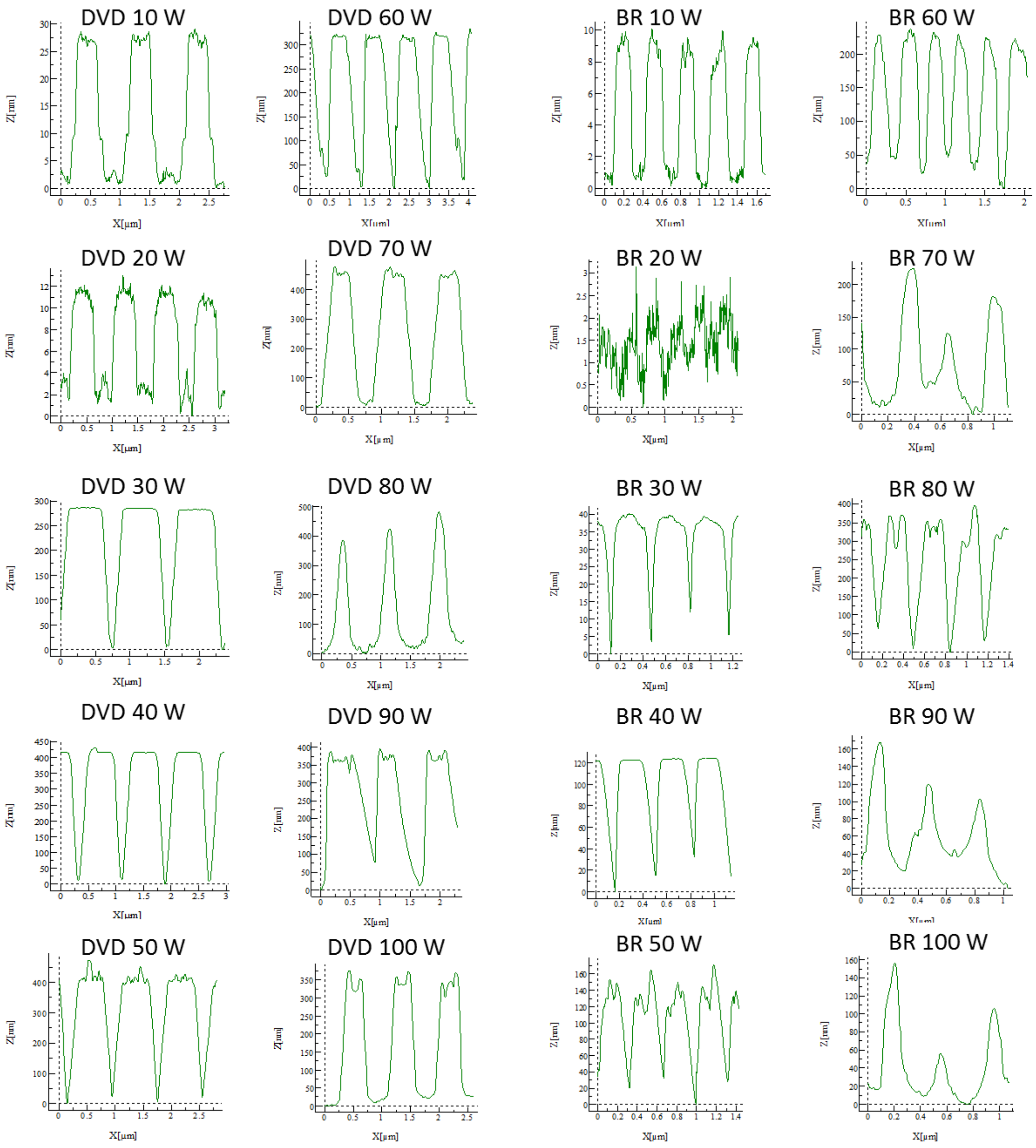

Figure S2. AFM profiles after RIE with SF_6/O_2 mixture plasma for different RIE powers.

Calculation of Wenzel model and comparison with our contact angle.

In order to discern the wetting state of the fabricated samples (Wenzel, Cassie-Baxter state or in the transition state) we have calculated the critical angle (θ_{cri}). The critical angle was calculated using the equation: $\cos\theta_{cri} = \frac{f-1}{r-f}$ where f is is the fraction of solid/liquid interface and r is the roughness factor. The roughness factor is defined as the actual surface to the geometric surface. It is a measure of how surface roughness affects a homogeneous surface. Roughness factor can be calculated from the geometrical parameters of the profiles measured by AFM. Tables S1 and S2

show the critical contact angle obtained for DVD-PDMS and BR-hPDMS samples and the geometrical dimensions (Figure S3) used for the calculation.

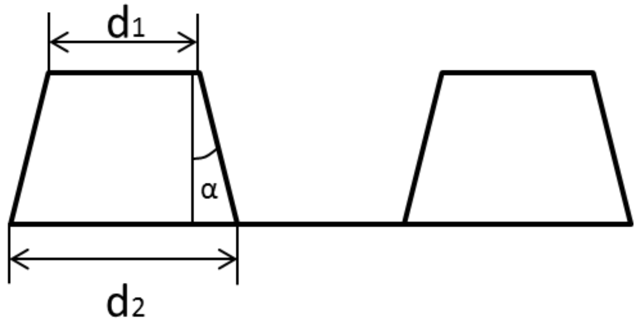

Figure S3. Schematic illustration of the geometrical dimensions used to calculate the wetting state and critical angle.

Table S1. Dimensions of DVD-PDMS samples.

Power (W)	α (°)	d_1	d_2	r	f	Θ_{cri}
100	11.9	240	473	2.84	-	-
90	19.81	310	711	2.94	0.51	101
80	12.96	300	593	3.06	-	-
70	14.81	368	700	3.09	0.48	100
60	14.6	293	740	2.34	-	-
50	34.99	289	652	2.81	0.54	101
40	29.44	464	622	2.49	0.39	106
30	62.7	450	755	2.24	0.35	109
20	87.25	415	657	1.44	0.75	111
10	80.72	388	644	1.35	0.79	111

Table S2. Dimensions of BR-hPDMS samples.

Power (W)	α (°)	d_1	d_2	r	f	Θ_{cri}
100	11.9	71	151	3.09	0.46	101
90	19.81	50	161	2.66	0.52	102
80	12.96	108	245	4.41	-	-
70	14.81	63	182	3.56	0.42	100
60	14.6	179	302	3.64	0.44	99
50	34.99	197	295	1.75	0.75	104
40	29.44	152	267	2.09	0.63	104
30	62.7	187	342	1.61	0.55	114
20	87.25	157	262	1.32	0.85	107
10	80.72	138	211	1.23	0.88	109

The Wenzel model is valid between θ_{cri} and $\pi/2$. If we compare our experimental contact angle (Table S3) with the θ_{cri} we can confirm that our the measured wetting states are Wenzel (Figure S4).

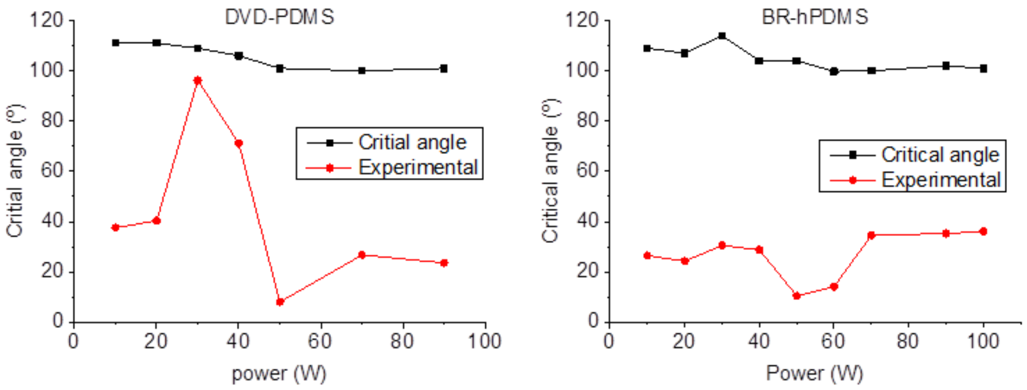

Figure S4. Critical and measurement angles vs. RIE power for DVD-PDMS and BR-hPDMS samples.

The Wenzel model describes the homogeneous wetting regime (Figure S5) and is defined by the following equation for the contact angle on a rough surface: $\cos\theta^* = r\cos\theta$, where $\cos\theta^*$ is the apparent contact angle that corresponds to the stable equilibrium state and r is the roughness factor. The angle θ is the Young contact angle as defined for an ideal surface.

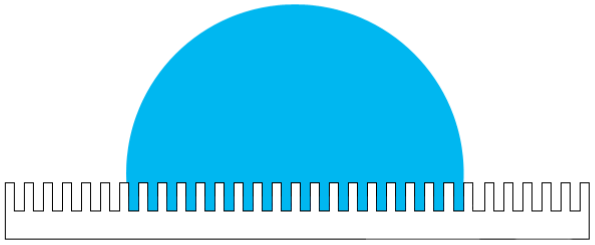

Figure S5. Wenzel model (https://en.wikipedia.org/wiki/Wetting).

The Table S3 show the theoretical and experimental contact angles obtained for DVD-PDMS and BR-hPMDS samples.

Table S3. Theoretical and experimental contact angles for DVD-PDMS and BR-hPDMS samples.

	Wenzel model (º)		Experimental (º)	
Power (w)	DVD-PDMS	BR-hPDMS	DVD-PDMS	BR-hPDMS
100	70.97	72.54	-	36.1
90	71.63	70.12	23.6	35.3
80	72.36	77.87	-	-
70	72.54	74.87	26.8	34.6
60	67	75.28	-	14.2
50	70.73	58.06	8.1	10.5
40	68.1	63.89	71.2	28.8
30	65.79	55.24	96.2	30.6
20	49.8	43.36	40.4	24.3
10	46.52	41.41	37.6	26.5

Figure S6 shows the graphical representation of the Wenzel angles and the experimental contact angles vs. power RIE for the different sets of samples.

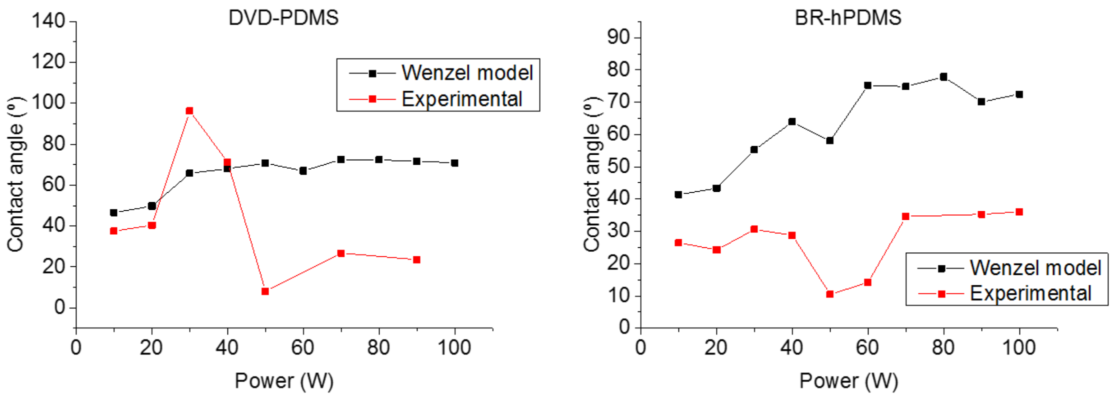

Figure S6. Theoretical and experimental contact angle for DVD-PDMS and BR-hPDMS samples.

Theoretical and experimental comparison of reflectivity.

Optical simulations were performed for the reflectance of the nanowire surfaces. The reflection of the different diffraction gratings has been computed by means of the Fourier Modal Method [1]. Each structure is defined by a period, filling factor or linewidth and groove depth according to the values reported in Tables 1 and 2. The refractive index of silicon was taken from Green and Keevers [2]. The simulations showed a strong peak of reflectivity for light with s-polarization at 280 nm, a wavelength very relevant for the detection and quantification of peptides and aminoacids by UV optical absorbance. Silicon has a maximum of reflectivity around this wavelength, but the reflectance peak is wide. The simulations show that the nanowire surface produces a grating effect that narrows the 280 nm peak in comparison with the reflectance of the silicon substrate without any pattern. The narrowing helps to spectrally isolate or filter other wavelengths that can disturb the optical detection by UV absorbance or other means. Our experimental measurements confirm the presence of the 280 nm peak and the narrowing effect by the grating of Si nanowires. The results show also that for all of the nanowire surfaces the peak at 280 nm appears at a spectral position that is robust against the change of the shape of the surface (i.e., height and width of the nanowires) for both sets of samples, although somehow better for DVD-patterned samples.

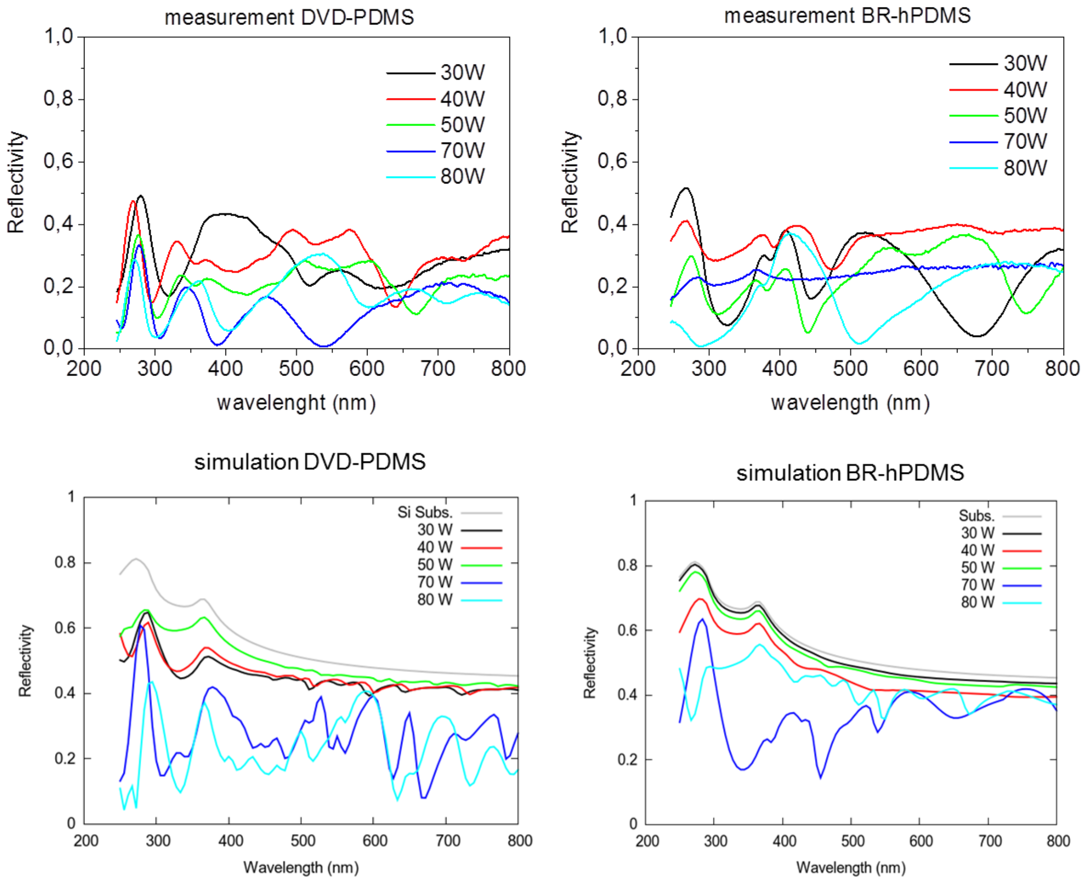

Figure S7. Theoretical and experimental s-polarized reflectivity for DVD-PDMS and BR-hPDMS samples.

References

[1] V. Liu and S. Fan, “S4 : A free electromagnetic solver for layered periodic structures,” *Computer Physics Communications*, **183**, 2233–2244, (2012)

[2] M. A. Green and M. J. Keevers, "Optical properties of intrinsic silicon at 300 K", *Progress in photovoltaics* **3**, 189-192 (1995)
